# Follow-Up Support for Effective type 1 Diabetes self-management (The FUSED Model): A systematic review and meta-ethnography of the barriers, facilitators and recommendations for sustaining self-management skills after attending a structured education programme

**DOI:** 10.1186/s12913-018-3655-z

**Published:** 2018-11-27

**Authors:** Fiona Campbell, Julia Lawton, David Rankin, Mark Clowes, Elizabeth Coates, Simon Heller, Nicole de Zoysa, Jackie Elliott, Jenna P. Breckenridge

**Affiliations:** 1 School of Health and Related Research (ScHARR), University of Sheffield, Regent Court, 30 Regent Street, S1 4DA Sheffield, England; 2 The Usher Institute of Population Health Sciences and Informatics, Edinburgh Medical School of Molecular, Genetic and Population Health Sciences, University of Edinburgh, Teviot Place, Edinburgh, EH8 9AG Scotland; 3 Diabetes Centre, King’s College Hospital, Denmark Hill, London, SE5 9RS England; 4 Sheffield University School of Medicine, Academic Unit of Diabetes, Endocrinology, and Metabolism, School of Medicine and Biomedical Sciences, Sheffield, UK; 5 School of Nursing and Health Sciences, University of Dundee, 11 Airlie Place, Dundee, DD1 4HJ Scotland

**Keywords:** Type 1 diabetes, Self-management, DAFNE, Structured education, Meta-ethnography, Qualitative evidence synthesis, FUSED

## Abstract

**Background:**

People with type 1 diabetes who attend structured education training in self-management using flexible intensive therapy achieve improved blood glucose control and experience fewer episodes of severe hypoglycaemia. However, many struggle to sustain these improvements over time. To inform the design of more effective follow-up support we undertook a review of qualitative studies which have identified factors that influence and inform participants’ self-management behaviours after attending structured education and their need for support to sustain improvements in glycaemic control.

**Methods:**

We undertook a meta-ethnography of relevant qualitative studies, identified using systematic search methods. Studies were included which focused on participants’ experiences of self-managing type 1 diabetes after attending structured education which incorporated training in flexible intensive insulin therapy. A line of argument approach was used to synthesise the findings.

**Results:**

The search identified 18 papers from six studies. The studies included were judged to be of high methodological quality. The line of argument synthesis developed the **F**ollow-**U**p **S**upport for **E**ffective type 1 **D**iabetes self-management (FUSED) model. This model outlines the challenges participants encounter in maintaining diabetes self-management practices after attending structured education, and describes how participants try to address these barriers by adapting, simplifying or personalising the self-management approaches they have learned. To help participants maintain the skills taught during courses, the FUSED model presents ten recommendations abstracted from the included papers to provide a logic model for a programme of individualised and responsive follow-up support.

**Conclusions:**

This meta-ethnography highlights how providing skills training using structured education to people with type 1 diabetes does not necessarily result in participants adopting and sustaining recommended changes in behaviour. To help people sustain diabetes self-management skills after attending structured education, it is recommended that support be provided over the longer-term by appropriately trained healthcare professionals which is responsive to individuals’ needs. Although developed to inform support for people with type 1 diabetes, the FUSED model provides a framework that could also be applied to support individuals with other long term conditions which require complex self-management skills to be learned and sustained over time.

**Trial registration:**

PROSPERO registration: CRD42017067961.

**Electronic supplementary material:**

The online version of this article (10.1186/s12913-018-3655-z) contains supplementary material, which is available to authorized users.

## Background

Type 1 diabetes mellitus (T1DM) is a chronic metabolic disease, characterized by hyperglycemia, which develops when the pancreas stops producing insulin due to auto-immune destruction of the β cells [1]. Global incidence rates are increasing at approximately 3–4% per year [2]. If not managed optimally, T1DM can lead to an increased risk of cardiovascular disease, microvascular complications [3] and premature death [4, 5]. Reaching and maintaining the glucose levels necessary to prevent complications requires the acquisition of a number of complex skills. Achieving these glucose targets also brings with it an increased risk of hypoglycaemia due to the limitations of subcutaneous insulin delivery. As a result, many people struggle to achieve optimal glycaemic control; hence, diabetes self-management education/training is now considered a critical element of care [6].

Education programmes for people with diabetes have evolved from didactic approaches, where knowledge is imparted by health professionals, to theoretically-informed structured education programmes (SEPs) incorporating group interaction, experiential learning, skills-based training and problem-solving in order to promote self-care [7, 8]. Concurrent with these developments, more flexible approaches to diabetes self-management have been promoted. This includes flexible intensive insulin therapy (FIIT) which is now widely recommended for type 1 diabetes self-management. FIIT comprises long-acting basal insulin injected once or twice daily, and bolus doses of quick acting insulin adjusted to take into account the carbohydrate content of snacks and meals. Individuals using FIIT are advised to perform regular self-monitoring of blood glucose levels (normally undertaken pre-meal and pre-bed). To calculate appropriate quick acting insulin doses, they are taught how to estimate the carbohydrate content of meals and snacks and, using ratios, to adjust doses in ways which also take account of their current blood glucose reading and the results of previous tests. To help people achieve clinically recommended blood glucose target ranges, they are also given instruction on how to use corrective doses of insulin to counter high blood glucose readings and how to consume carbohydrate in the correct amounts to address low blood glucose. Instruction is also given on how to interpret patterns and/or changes in blood glucose readings collected over time to ascertain whether to adjust mealtime ratios or basal insulin doses in order to maintain blood glucose within clinically-recommended target ranges [9, 10].

Developed in Düsseldorf, at the Diabetes Treatment and Teaching Programme (DTTP) [11], FIIT is a fundamental component of SEPs in countries around the world [12], many of which are based on the Düsseldorf model [11]. This includes the Dose Adjustment for Normal Eating (DAFNE) programme [13, 14], which is recommended for all adults with T1DM in the UK [15]. Research exploring DAFNE, and other SEPs for people with T1DM has reported short and medium term improvements in HbA1c and quality of life, and reductions in incidence of severe hypoglycaemia [13, 16–21]. However, while improvements in quality of life are generally maintained, people frequently experience a decline in their glycaemic control over time, which suggests they are unable to sustain FIIT self-management practices in the long-term [18, 21, 22]. The reasons for declines in glycaemic control are poorly understood [23], which has prompted calls for research to be undertaken to better understand participants’ experiences after attending SEPs [14]. This has led to various studies being undertaken to better understand the challenges people with T1DM encounter when sustaining a FIIT approach following SEPs and to identify their longer-term information and support needs. However, to our knowledge, there have been no reviews of studies exploring participants’ experiences after FIIT training to help them maintain skills taught during SEPs and sustain improvements in glycaemic control. To design effective follow-up support, it is necessary to identify factors that influence and inform self-management behaviours in people with T1DM following participation in a SEP, and their experiences of follow-up support and how this might be improved.

## Methods

The aim of this paper was to a) synthesise from existing qualitative literature, the experiences and views of people with T1DM about sustaining learning and self-management skills after attending a SEP providing training in FIIT and b) to identify recommendations for follow-up support provision. The work comprised the first phase of a larger study to develop and trial a new structured education programme and follow-up support package for people with T1DM, called ‘DAFNE*plus*’ [24]. We sought to develop an evidence informed model to guide intervention design, setting out to address the following objectives

1. To describe the scope, nature and quality of the qualitative research exploring how people with T1DM apply and sustain learning and self-management skills after attending a SEP.
2. To identify the barriers and facilitators to applying and sustaining learning and self-management skills after attending a SEP.
3. To explore what follow-up support has been provided, how acceptable it has been and whether it helped people with T1DM to apply and sustain learning and self-management skills after attending a SEP.
4. To identify what recommendations have been made about follow-up support provision to best support people with T1DM to apply and sustain self-management skills taught on a SEP.

We employed Noblit and Hare’s [25] seven stage meta-ethnographic approach as this has been used successfully by others to develop theory to underpin practice interventions [26]. Meta-ethnography goes beyond describing or summarising literature, to deriving new interpretations that are grounded in, but extend beyond, individual empirical studies [27]. Meta-ethnography seeks to develop a coherent theory that is economical (it explains what is going on in the literature in the simplest way), cogent (the explanation is achieved without redundancy, ambiguity and contradiction), and credible (it is useful and relevant for the intended audience) [25]. Therefore, in addition to offering new conceptual insights to inform the wider type 1 diabetes evidence base, we anticipated that a meta-ethnographic approach would enable us to directly inform design and evaluation of a new follow-up intervention to be delivered as part of the ‘DAFNE*plus*’ study.

### Stage 1 and 2: Getting started and deciding what is relevant

Stages 1 and 2 of the meta-ethnographic approach involved establishing the scope of the synthesis and locating relevant studies [25]. We conducted a systematic search to identify papers that presented empirical qualitative data about the experiences of adults with T1DM after attending a SEP. As meta-ethnography does not aim to summarise an entire body of knowledge or to draw any statistical inference, it does not necessarily pursue exhaustive search strategies typical of quantitative systematic reviews [28]. However, to identify the scope and nature of qualitative studies published about the topic (objective 1), identify any gaps in the existing evidence base and ensure that recommendations for follow-up support were drawn from all available, relevant evidence we opted to utilise a systematic search strategy.

#### Search strategy

Databases searched included MEDLINE, Embase, CINAHL, PsycINFO and Web of Science using a combination of subject terms and keywords, which were developed in discussion with topic experts and informed by existing literature. We used the SPIDER model [29] to support development of our search strategy (Table 1). Where available, validated search filters (developed by the HEDGES team at McMaster University) were used to restrict results to qualitative evidence [30]. We supplemented the database searches by checking the reference lists of included papers, contacting key authors, and citation tracking. Where no validated qualitative filter was available (e.g. in Web of Science), qualitative terms drawn from the Medline filters were used to search within the database. Searches used a combination of subject headings and free text terms occurring in titles and abstracts. The Medline strategy was developed through several iterations in consultation with the larger ‘DAFNE*plus*’ project team, and also validated against known relevant articles. This search was then used as a template which was modified when searching other databases. Searches were limited to 1978 onwards to coincide with the development of the first SEP for T1DM [11]. Papers were limited to English language for pragmatic reasons. An example of our search strategy is included in Additional file 1.

**Table 1:** Terms used to develop search strategy

Sample:	Adults with type 1 diabetes
Phenomenon of Interest:	Diabetes self-management education, patient education, patient information, structured education, specific names (e.g. DAFNE, BERTIE), follow up support, health professional support, flexible intensive insulin therapy (FIIT), technology
Design:	Grounded theory, phenomenology, ethnography, interview, focus group, observation, questionnaire, thematic analysis, constant comparison, content analysis, themes, category, experience, views, perspectives
Evaluation/outcomes:	Sustained behaviour change, self-management skills, BG control, glycaemic control, BG targets, BG monitor, carb counting, hypo management, insulin dose adjustment, diet choices, mealtime ratios, confidence, coping
Research:	Qualitative studies

#### Screening and selection

Titles and abstracts were screened against the inclusion and exclusion criteria (see Table 2) by two members of the review team independently. For abstracts that met the inclusion criteria, papers were retrieved in full and re-assessed against the inclusion criteria by two members of the research team to agree on final inclusion.

**Table 2:** Inclusion and Exclusion Criteria

Inclusion	
• Papers reporting qualitative data that captures the experiences of adults with T1DM after attending a SEP teaching FIIT, and that includes data relevant to understanding the barriers and facilitators to sustaining self-management skills following the SEP	
• Published after 1978.	
Exclusion	
• Papers that present quantitative data only.	
• Papers that are not explicitly about T1DM (e.g. papers where participants may have type 1 OR type 2, or papers which focus on other long term conditions).	
• Papers presenting data about SEPS that are not based on FIIT.	
• Citations with results only published as conference abstracts	
• Published in languages other than English	

We observed that several papers were derived from the same studies and based on the same dataset. On appraisal of the full texts, we identified that, rather than repeating the same findings, each paper offered a distinct focus and presented different aspects of the data. We therefore took all papers forward for inclusion in recognition that they may each contribute to the synthesis in conceptually different ways.

**Fig. 1:**
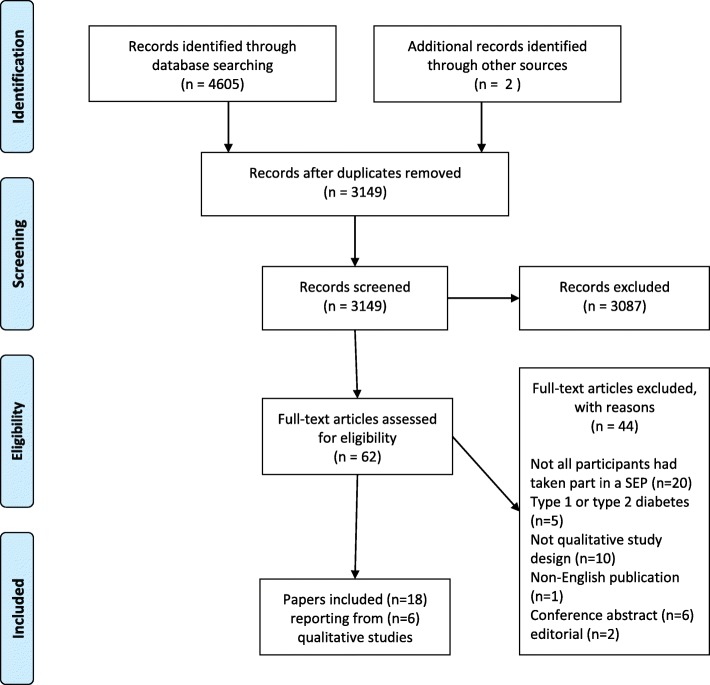
PRISMA flow diagram

Figure 1 provides details of the screening and selection process, as recommended by the Preferred Reporting Items for Systematic Reviews and Meta-Analyses (PRISMA) statement (http://www.prisma-statement.org). We also used guidance from the eMERGe website to inform the reporting of this meta-ethnography. [31]

**Fig. 2:**
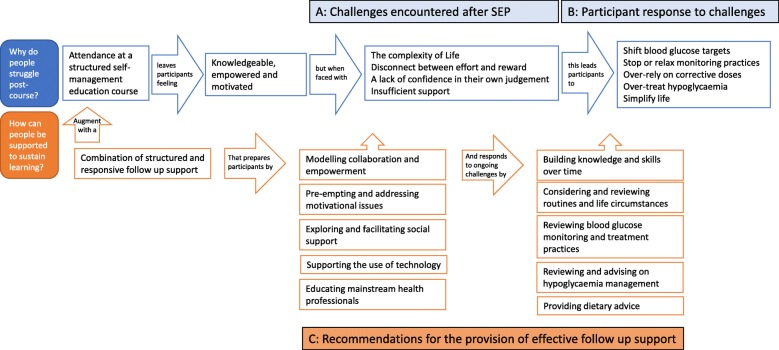
Diagrammatic Representation of the Line of Argument: The FUSED model

### Stage 3: Reading the studies

This stage involved familiarising ourselves with the content and context of the included papers and extracting information about key study characteristics [25, 32, 33]. Details were extracted by two authors working independently and were documented by each in a separate version of a purpose-designed matrix under the following headings: author, year, country, aims, description of intervention, participants, data collection, data analysis, and presentation of findings. Both matrices were compared and combined to ensure that all relevant characteristics were identified and described. Reading the studies also included quality appraisal, guided by the current version of the NICE (2012) manual procedures for assessment of qualitative studies [34]. Both reviewers agreed the combined assessment of each study. All studies, regardless of quality, were included in the review, with the expectation that the poorer quality studies would contribute less to the synthesis [25]. Appraising the included studies ensured careful and systematic reading of the included studies [27] and yielded descriptive information on aspects of the quality of the included papers which provided context for the meta-ethnographic synthesis. 

### Stage 4: Determining how the studies are related

This stage involved exploration of the relationships between the papers and identifying common concepts shared between them [25, 32, 33]. It involved making ‘a list of key metaphors, phrases, ideas and/or concepts (and their relations) used in each account’ [25]. Within meta-ethnography, ‘metaphors’ or ‘concepts’ refer to the meaningful, explanatory ideas expressed within the included papers. We operationalised this process by extracting, from each paper, all statements of key findings (from the findings/results sections of included papers) and statements of key recommendations (from the discussion and conclusion sections of included papers). Using an Excel spreadsheet for each paper, with the headings ‘key findings’ and ‘recommendations’, we listed the key statements from each paper. Statements were formed from either the authors’ own words (where concepts were briefly and clearly described in the original paper), or from paraphrasing (where descriptions were unclear and/or lengthy).

### Stage 5: Translating the studies into one another

This stage involved directly and systematically comparing concepts across individual papers [28, 35]. We compared each statement of key findings extracted in stage 4 and grouped them according to similarity, arriving at a series of higher level concepts. We then used this same process of comparison for the statements of recommendation, looking for similarities and differences and arriving at a higher level set of concepts. We purposefully kept the statements regarding findings and recommendations separate it order to distinguish between concepts arising directly from participant data and those developed by authors in response to the findings.

### Stage 6: Synthesising translations

Noblit & Hare [25] proposed three approaches to synthesis, depending on how the concepts from each paper relate to one another: *reciprocal syntheses* (where papers are conceptually similar), *refutational syntheses* (where papers contradict one another), *line of argument synthesis* (where papers identify different parts of the whole story). We identified that a line of argument synthesis would be most suitable because, while there was overlap between the papers, they each dealt with a slightly different aspect of self-management. There were no refutational findings in the included papers. The higher level concepts generated during stage 5 were systematically compared to explore how they related to one another, resulting in a coherent, logical, explanatory model. This was an iterative process until the concepts fitted together without overlap or redundancy. In addition, we also looked for and explained the inter-connections between key findings and recommendations to arrive at an integrated explanation.

### Stage 7: Expressing the synthesis

The resultant line of argument takes the form of a logic model. The model provides a high level explanation about why people struggle to sustain self-management practices following attendance at a structured education programme and outlines the essential elements of effective follow-up support.

## Results

### Included papers

The search identified 3149 citations and following screening 64 full text papers were retrieved. A further 46 papers were excluded on reading the full text. The list of excluded studies and reason for exclusion is given in Additional file 2. Eighteen papers, reporting findings from six studies, were included in the synthesis [36–53]. Three presented findings from a longitudinal interview study involving 40 individuals who took part in a RCT in Ireland which compared group based follow-up with usual care after attending a DAFNE course [36–38]. One of these papers used mixed-methods [38], however, the qualitative data were of sufficient depth and quality for inclusion. Nine papers reported findings from a longitudinal interview study with 30 individuals after attending a DAFNE course in the UK [39–47]. Two papers presented findings from repeat interviews with 42 participants in the Relative Effectiveness of Pumps over Multiple Daily Injections and Structured Education (REPOSE) trial in the UK, where participants in both arms initially attended a DAFNE course [48, 49]. Two papers presented findings from a UK-based interview study involving 21 participants who had attended a DAFNE course between 3 months and 11 years previously [50, 51]. One paper presented findings from an interview study with 24 participants who had taken part in the DAFNE-HART course, which was specifically developed for people with impaired awareness of hypoglycaemia [52]. One paper presented findings from a qualitative study of seven participants’ experiences of using a mobile phone app after attending a DAFNE course [53]. Table 3 provides an overview of each of the six primary studies, detailing the participants and the type of structured education and follow-up support they had received. Table 4 provides detailed information about each of the papers included in the synthesis and Table 5 presents the results of the quality assesment of each included paper.

**Table 3:** Overview of the six primary studies: participants, types of structured education and follow up interventions studied

Study title, funder, location	Study aim	Participants	Nature of the structured education studied	Nature of the follow-up provision studied
Irish DAFNE Study Ireland [36–38]	A cluster randomised trial to compare the outcomes for patients receiving individual routine care with those receiving additional group based follow up support.	Purposive sample (*n* = 40) from 5 DAFNE centres, making up approximately 10% of the total study sample (*n* = 437). Years since diagnosis ranging from 2 to 31+ years, Gender: 15 males, 25 females. Most participants under the age of 50, with 5 between the ages of 51–70 and most (27.5%) in the 10–20 year bracket. Socioeconomic background not reported.	DAFNE course delivered over 5 consecutive days.	Group follow-up was delivered at 6 and 12 months post-course using a curriculum designed for the study. Group follow up was delivered by the same DAFNE educators who delivered the course and reviewed progress and goals. Participants in the control arm received individual support from health professionals in diabetes clinics as part of routine care.
UK DAFNE study UK [39–47]	A qualitative study exploring barriers and facilitators to sustaining a FITT approach following course attendance and over time to provide insight into why some people cannot sustain intensive self-management.	Patients (*n* = 30) from UK DAFNE centres (*n* = 5). Recruited using an opt-in procedure, with purposive sampling of participants from the last two courses to ensure diversity. Years since diagnosis ranging from 1 to 45 years. Gender: 16 women, 14 men Ages ranged from18-56 yrs. Occupation: 30% professional 36.7% semiskilled, 20% unskilled, 10% students,3.3% unemployed	Participants attended standard DAFNE courses delivered over 5 consecutive days	Post course, patients received routine clinical care, either in hospital or general practice, provided by health professionals from whom they received clinical care and reviews prior to DAFNE. Patients also received DAFNE educators’ contact details and were invited to contact them if they had questions/concerns. Patients were given the opportunity to attend a group-based, half-day follow up session six weeks post course, facilitated by their course educators and involving their fellow course attendees. Some DAFNE centres offered a further group session at 6 or 12 months post course.
REPOSE (Relative Effectiveness of Pumps Over MDI and Structured Education) UK [48, 49]	A randomised controlled trial to establish the added benefit of a pump over multiple injections on glycaemic control and hypoglyceamia in individuals with Type 1 diabetes receiving similar high quality structured training.	Purposive sample (*n* = 42) of participants from seven REPOSE centres. 23 participants using a pump, 19 using MDI. 36 participants were using bolus advisors at baseline, with 32 still using them 6 months later. Gender: 20 female, 22 male Ages ranged from 24 to 66 and Occupation: 31% had a professional occupation, 36% were semi-skilled, 9.5% unskilled, 7% were students, and 16.5% were unemployed.	Participants in both trial arms attended a DAFNE course over 5 consecutive days. Participants in both arms were taught how to use bolus advisors during the course.	Routine care was provided to all participants from their usual healthcare providers. Additionally, participants were required to attend appointments at 6, 12 and 24 months in order for data to be collected for the trial. DAFNE educators were also available at these time points to provide advice and respond to any issues.
DAFNE UK [50, 51]	Qualitative patient led study of experiences of diabetes structured education.	Purposive sample (*n* = 21) from three established UK DAFNE centres. The ‘new student group’ (*n* = 11) attended DAFNE during the study dates. The ‘graduate group’ (*n* = 10) had taken part in DAFNE between 3 and 11 years previously (mean of 7). 10 had been diagnosed as children or teenagers, and 11 as adults Gender: 12 female, 9 male Ages ranged from 20s–60s Employment: 12 were in full time employment, 3 part-time, 2 students, 2 on sick leave and 2 retiresd. Ethnicity: 17 were White British, 1 White other, 3 Black Caribbean	All participants had attended a standard DAFNE course delivered over 5 consecutive days.	Routine care and usual support provided following standard DAFNE courses, which can differ across sites.
DAFNE-HART (Hypoglycaemia Awareness Restoration Therapy) UK [52]	To develop and carry out a qualitative evaluation of the DAFNE-HART intervention for DAFNE graduates with ongoing problematic hypoglycaemia.	24 adults with problematic hypoglycaemia (2 or more episodes of severe hypoglycaemia, requiring 3rd party assistance since they first completed standard DAFNE), and a Gold score of 4 or more, were recruited from two UK DAFNE centres to attend a DAFNE-HART course. 21 of the 24 participants took part in the qualitative evaluation. Years since diagnosis: mean 31.2 (SD 11.3 years) Gender: 9 female, 15 male Age: mean 53.3 (SD 8.1 years) Occupation not reported.	All participants had previously attended a standard DAFNE course but still had persistent impaired awareness of hypoglycaemia.	The course was delivered over 6 weeks (3 full day group sessions in weeks 1–3, individual face-to-face and phone appointments in weeks 4–5, a full day session with invited significant others in week 6). The programme revised DAFNE principles in relation to hypoglycaemia and drew upon cognitive behavioural therapy and motivational interviewing to address the motivational and perceptual barriers to restoring hypoglycaemia awareness. Participants received a workbook and a personalised ‘hypo-prevention plan’.
RapidCalc Australia [53]	Qualitative interview study to obtain user feedback on the usability of a phone based bolus calculator application called ‘RapidCalc’.	Graduates of a DAFNE course (*n* = 7) who had completed a standard DAFNE course within the past 13 months. Years since diagnosis: mean 27 years. Gender: 5 female, 2 male Age: mean 36 years	DAFNE graduates given the RapidCalc app, which enables: bolus dose determination; diabetes diary recording; report generation; communication with health professionals. Participants received one group based app education session with the app developer a diabetes educator who programmed the app with each individuals’ insulin adjustment algorithms.	Participants were invited to contact the diabetes educator for assistance with dose adjustment at any time between the app education session and taking part in the focus group one month later.

**Table 4:** Summary of Qualitative Papers included in the synthesis

Paper	Aim	Sample	Data collection	Data analysis	Presentation of findings
Casey et al. 2011 [36]	To identify key factors impacting on patients’ ability to assimilate DAFNE principles into their daily lives and how and why these may change over time.	Purposive sample (*n* = 40) from 5 DAFNE centres.	Paper presents data from semi-structured interviews carried out by two researchers over a 36 month period during 2006–2009. Participants were interviewed at three different time periods, 6–8 weeks, 6 months and 12 months post attendance at DAFNE. 2 participants were lost to follow up at the 12 month interview due to illness. Total interviews =118. Data gathered over a 36 month period from 2006 to 2009	Longitudinal analysis, involving three stages: 1. analysis of specific time points using thematic analysis and axial coding to identify categories; 2. cross sectional analysis to look for change between time points in individual cases; 3. thematic analysis of changes over time.	Four themes: 1. embedded knowledge 2. continued responsive support 3. enduring motivation 4. being empowered
Murphy et al. 2011 [37]	To understand the experience of participants in the DAFNE programme and identify factors that influence participants’ implementation of self-management guidelines.	Same participants as paper [36].	Same dataset as paper [36], but only presents data collected within the first 24 months.	Constant comparative analysis undertaken concurrently with data collection. Open coding, axial coding and development of a core category.	Developed a core category of “being in control” which included 5 factors - knowledge, motivation, support, relationship shift and empowerment.
Casey et al. 2016 [38]	To explain the factors affecting glycaemic control, measured by HbA1c, following completion of a DAFNE course.	Same participants as paper [36, 37], but 6 participants were lost to follow up (1 man, 5 women) giving a total of *n* = 34.	Same dataset as paper [36] but with the addition of quantitative measures - HAD, DSQOLS,PAID, clinical data on HbA1c	As for paper [36] but findings were merged with the quantitative data set to show how participants’ perspectives on their self-management (the themes from paper [36]) linked to their quantitative outcomes.	Findings presented according to three groups of participants: 1. out of control (*n* = 17, above 8.0% at baseline and at 12 months) 2. Out of control but getting control (*n* = 6, above 8.0% at baseline, but reduced to less 12 m) 3. In control and remained in control (*n* = 5, started below 8.0% and remained under).
Lawton & Rankin 2010 [39]	To establish how, and why, structured educational programmes work to foster changes in diabetes self-management practices	Patients (*n* = 30) from UK DAFNE centres (*n* = 5). Recruited using an opt-in procedure, with purposive sampling of participants from the last two courses.	Open, unstructured observation of DAFNE courses and in-depth interviews with participants lasting approximately 1 hour and carried out within one week of course completion. Patient interview topic guides were individually tailored based on observational data and included the following: history of their diabetes and health service contact prior to DAFNE, their experiences of managing diabetes over time, their motivations for attending DAFNE, any changes they had made to their insulin doses and other self-management practices post-course, and their views about how future courses could be improved. Data collected between June 2008 and February 2009.	Iterative thematic analysis informed by social constructivist position. Data were analysed by two independent researchers, then cross-compared to explore interviewees’ underlying reasoning, discuss deviant cases, and resolve any differences in interpretation, and to reach an agreement on recurring themes. Data were organised into higher codes, “risk thermostat” was used as a conceptual starting point for the analysis.	Eight themes: 1. Coming together in a group - overcoming isolation 2. The group based approach - enhanced learning 3. Life before DAFNE - cocooning and risk avoidance 4. Doing reviews - becoming a dose-adjusting subject 5. Supervised risk-taking 6. Recalibrating risk thermostats - drawing upon others’ experiences 7. Surveillance and shepherding 8. Rules and accountability.
Lawton et al. 2011 [40]	To explore whether and why people with T1D change their food and eating practices in light of the FIIT training during a five day DAFNE course, and whether, and why, any dietary changes made attenuated or were sustained over time.	Same participants as paper [39].	Same dataset as paper [39] but with the addition of further semi-structured interviews with patients at 6 and 12 months post-course, each lasting between 1 and 2 hours. Topic guides built on previous data collection. Data presented in this paper were elicited through questions about how and what ways participants’ perceptions and conceptualisations of food had changed as a result of FIIT conversion and the requirement to match insulin to carbohydrates. Data collected between July 2008 and February 2010.	Same approach as paper [39] with the addition of longitudinal analysis to explore continuities and changes in participants’ dietary choice and eating practices over time, and the reasons for these. Within respondent accounts were also compared and contrasted between respondent accounts, enabling identification of overarching themes.	Three themes: 1. Initial expectations and actual experiences of managing one’s diet using FIIT 2. Reasons for dietary continuity *Subthemes: routinisation/ habituation; a lack of interest in food; eating on FIIT - freedom with new restrictions* 3. Changing perceptions of food
Rankin et al. 2011 [41]	To examine whether, and how, patients made use of FIIT practices and if these practices were sustained, to inform the development of effective long-term support for patients converted to flexible intensive insulin therapy.	Same participants as paper [39].	Same dataset as paper [40] Data presented in this paper were elicited through questions about patients’ experiences of diabetes self-management prior to and after attending DAFNE, their contact with health services, everyday work/home lives and other issues affecting self-care.	Same approach as paper [40] but with a specific focus on how FIIT had been implemented, whether, how and why this approach had changed or been sustained over time, and patients’ views about the advantages, disadvantages, ease and difficulties of integrating this regimen within their everyday lives.	Three themes: 1. Commitment to FIIT 2. The importance of routines 3. Making FIIT therapy work - restricting and making adjustments to lifestyles
Lawton et al. 2012 [42]	To explore patients’ experiences of, and views about, making adjustments to their insulin doses following the completion of a DAFNE course and over time.	Same participants as paper [39].	Same dataset as paper [40]. Data presented in the paper were elicited through questions about the factors and considerations informing the kinds of dose adjustments made (or not made) post-course and over time.	Same approach as paper [40].	Seven themes: 1. Experiences of adjusting quick acting insulin doses 2. Experiences of altering background insulin doses and mealtime ratios 3. Independent adjusters 4. Lack of confidence/ability - deference to health professionals 5. Lack of motivation and record keeping 6. Experiences of using corrective doses 7. Health service contact
Rankin et al. 2012a [43]	To explore patients’ experiences and views of implementing clinically recommended blood glucose targets after attending a structured education programme	Same participants as paper [39].	Same dataset as paper [40]. Data presented in the paper were elicited through questions about participants’ pre-course understanding of blood glucose readings, their views on implementing BG targets pre-course, their attainment and use of targets post-course, whether, in what ways and for what reasons, their approaches to using targets had changed.	Same approach as paper [40] with a specific focus on continuities and changes in use of targets over time, and the reasons for this.	Two themes: 1. Benefits of using targets post-course 2. Adapting and personalising targets over time *Subthemes: simplifying targets; fear of hypoglycaemia; mitigating feelings of failure; elevating targets to address hypoglycaemia unawareness; healthcare professionals’ roles in adjusting targets.*
Rankin et al. 2012b [44]	To explore patients’ experiences of and views about: their healthcare and the support they receive post-course; unmet support needs; and their recommendations for future healthcare support.	Same participants as paper [39].	Same dataset as paper [40]. Data presented in the paper were elicited through questions about patients’ views on their pre-course support, their post-course support needs, their experiences of attending structured follow up, their experiences of routine care, and their encounters with fellow course attendees and their views on any unmet support needs.	Same approach as paper [40].	Four themes: 1. Seeking reassurance and trouble-shooting opportunities; 2. Experience of seeking support from health professionals *Subthemes: healthcare professionals with relevant training; lack of support at routine hospital appointments; accessing educators* 3. Organised follow up meetings *Subthemes: attendance at group-based follow up; limitations of group based follow-up* 4. Provisions to address unmet needs.
Lawton et al. 2013 [45]	To explore participants’ experiences and views of self-treating hypoglycaemia and their reasons for following or not following course recommendations	Same participants as paper [39].	Same dataset as paper [40]. Data presented in the paper were elicited through questions about factors and considerations informing their approaches to treating mild hypoglycaemia, including reasons for changing their management approach over time.	As for paper [40].	Four themes: 1. Pre-course approaches to hypoglycaemia management 2. Reconfiguring practices in light of course attendance *Subthemes: panic and disorientation; a chance to indulge* 3. Developing and changing treatments strategies over time 4. Future support needs.
Rankin et al. 2014 [46]	To explore patients’ experiences of, views about and need for, social support post-course and over time. A key objective was to identify better ways to help patients use their social support networks to implement and sustain diabetes self-management practices.	Same participants as paper [39]. Additional information provided about patients’ domestic arrangements to provide context	Same dataset as paper [40]. Data presented in the paper were elicited through questions at baseline about the role of social support, participants’ perceived importance of support from family/friends/colleagues, and expectations for how social support may change over time. At follow up interviews, questions asked about if and how social support changed over time and what other forms of support participants would like to receive.	Same approach as paper [40].	Five themes: 1. Preferences for support *Subthemes: minimal involvement, auxiliary forms of support; monitoring and policing* 2. Post course changes to support 3. Parental support 4. Domestic and employment circumstances 5. Limitations of social support.
Heller et al. 2014 [47]	Research monograph reporting findings from a broader programme of research which included a qualitative study about the barriers to self-management post course and over time and patients’ views about how they could be better supported to sustain use of a flexible intensive insulin approach.	Qualitative part of the report presents data from same participants as paper [39].	Qualitative data from the same dataset as paper [40].	Qualitative data analysed using same approach as paper [40].	Qualitative data presented under two research questions: - what are patients’ experiences of self-management post-course and over time, and what barriers and facilitators do patients encounter? *5 themes: overview; habits and routines; food and eating practices; managing hypoglycaemia and using blood glucose targets; dose adjustments and health service contact.* - how could patients be better supported to sustain course learning/a FIIT approach over time?
Lawton et al. 2014 [48]	To explore participants’ experiences of using bolus advisors post course and over time and their likes and dislikes of this technology.	Purposive sample (*n* = 42) of participants from seven REPOSE centres.	Semi-structured interviews at baseline and 6 months (1 hour duration). Topic guides included: historical experiences of diabetes management and health service contact; perceived confidence/ability to undertake mathematical calculations; initial perceptions of bolus advisors; reasons for choosing/not choosing to use a bolus advisor; likes dislikes about the advisor; everyday experiences of using the advisor; reasons for following/not following recommended doses; perceived impact of the advisor on diabetes self-management; changes made to settings and parameters - by whom, how and why; information and support needed to facilitate effective use of advisors; recommendations for how advisor technology could be improved. Data was collected between November 2011 and April 2013.	Thematic analysis involving comparison of individuals’ baseline and follow up transcripts to identify continuities and changes in the use of bolus advisors over time, and the reasons for this. Longitudinal accounts were compared and contrasted across participants. NVivo9, a qualitative software package, was used to support analysis.	Three themes at baseline: 1. Motivations for and perceived benefits of using advisors 2. Initial experiences of using advisors 3. Calculating doses Two themes at follow up: 1. Dependency and deskilling; impact on disease self-management 2. Reasons for stopping/never starting. Themes cut across both MDI and pump users as the main issues and experiences reported by participants were found to be the same in both groups.
Heller et al. 2017 [49]	Research monograph reporting the outcomes of the REPOSE trial; this included embedded qualitative research which explored patients’ experiences in both arms of the trial (MDI vs pumps) to aid interpretation of trial outcomes.	Qualitative part of the report presents data from same participants as paper [48].	Qualitative data from the same dataset as paper [48].	Qualitative data analysed using same approach as paper [48].	Data presented in relation to cross-cutting improvements to quality of life, and as an aid to interpreting the quantitative quality of life data within the report.
Snow et al. 2013 [50]	To explore the impact of patient education on the lives of people with diabetes, including the effect on interactions with doctors and other healthcare professionals.	Purposive sample (*n* = 21) from three established UK DAFNE centres.	‘New students’ took part in narrative interviews 1 week pre-course and then 3 months post-course. They were also observed during the course. ‘Graduates’ took part in retrospective narrative interviews. This totalled 32 interviews and 146 hours of course observation. Interview topic guides, informed by a patient advisory group, asked open questions about ‘how you have learnt to manage diabetes’ with prompts where necessary to elicit data about interactions with others (e.g. friends, family, work colleagues, healthcare professionals of all kinds). DAFNE curriculum and patient facing course booklet were also treated as data. Data was collected between September 2011 to January 2012.	Transcripts and field notes were analysed using a mixture of thematic and structural narrative analysis. The ideal patient role being taught on DAFNE was compared with students’ personal life stories, and with their stories of others’ expectations of them once the course was over. Interviews were supplemented by observational data where they had told personal stories. Participant data was analysed separately and then cross-compared to elicit wider themes about how health professionals and lay people responded to their new status as DAFNE graduates.	Three themes: 1. Expectations - the role of expert patient 2. Experience - taking on the new role 3. Consequences - a clash of roles.
Snow et al. 2014 [51]	To explore the role of targets in teaching patient self-efficacy and self-management, including an exploration of the conflict between the ideal and the reality for people taking part in education.	Same participants as paper [50].	Same dataset as paper [50] but with the additional use of ‘touchpoints’ (participants were asked to describe a moment when diabetes management may have been explained to others at home, in the work place or with different types of health professionals).	Not described.	One theme - Reaching set HbA1c targets, with subthemes of ‘knowledge is power’, and ‘tricky but you can do it’
Shuttlewood et al. 2015 [52]	As part of a broader programme of research a qualitative study explored patients’ experiences of the DAFNE-HART intervention and its impact on how they managed and addressed impaired awareness of hypoglycaemia.	24 adults with ongoing problematic hypoglycaemia were recruited from two UK DAFNE centres to attend a DAFNE-HART course. 21 of the 24 participants took part in the qualitative evaluation.	In-depth telephone interviews conducted immediately post-course. Topic guides explored: participants’ reasons for agreeing to attend the course, how they felt about the group format, what aspects they liked or disliked, how they thought the course could be improved and the perceived impact the course had on their attitudes towards and experiences of preventing and managing hypoglycaemia. Dates of data collection not reported.	Inductive thematic analysis supported by NVivo. An initial coding frame was developed based on the research questions and a review of the literature and refined in response to concurrent data collection and analysis. Each transcript was read through several times to produce an initial description of the patterns in people’s experiences, i.e. the semantic themes. These were further organised into hierarchical themes, highlighting where experiences clustered and where they diverged.	Five themes: 1. changes in diabetes management 2. regaining cues 3. new diabetes-related cognitions 4. the dynamics of the programme – people and place 5. relationship with care provider
Knight et al. 2016 [54]	To obtain user feedback on the usability of the RapidCalc app in adults with T1DM already experienced in flexible MDI to inform further development of the app and identify user preferences.	Graduates (n = 7) who had completed a standard DAFNE course within the past 13 months.	Single focus group, lasting approximately 2 hours, held one month after being provided with, and educated in the use of, the RapidCalc app. The focus group was led by a diabetes educator unconnected with the development of the app and followed a semi-structured format, focusing on satisfaction, usability, learnability, efficiency, memorability, errors and future improvements. Dates of data collection not reported.	The focus group was transcribed verbatim and analysed by two independent researchers using thematic analysis. Themes were coded according to specific app features to highlight the features that participants found useful or not.	Three themes 1. bolus calculator features and trust 2. diary report features 3. satisfaction and control

**Table 5:** Quality Assessment of included papers

	Qualitative approachadequate	Study purpose clear	Study design defensible	Data collectionappropriate	Role of researcher clear	Context clear	Reliable methods	Rigorous data analysis	Rich data	Reliable analysis	Convincing findings	Relevant findings	Conclusions adequate	Ethical approval	Overall rating
Irish DAFNE															
Casey et al. (2011) [36]	Y	Y	Y	Y	Y	Y	Y	Y	Y	Y	Y	Y	Y	Y	provides details of the screening and selection process
Casey et al. (2016) [38]	Y	Y	Y	Y	Y	Y	Y	Y	Y	Y	Y	Y	Y	Y	++
Murphy et al. (2011) [37]	Y	Y	Y	Y	Y	Y	Y	Y	Y	Y	Y	Y	Y	Y	++
UK DAFNE															
Lawton et al. (2010) [39]	Y	Y	Y	Y	Y	Y	Y	Y	Y	U	Y	Y	Y	Y	++
Lawton et al. (2011) [40]	Y	Y	Y	Y	Y	Y	Y	Y	Y	U	Y	Y	Y	Y	++
Rankin (2011) [41]	Y	Y	Y	Y	Y	Y	Y	Y	Y	U	Y	Y	Y	Y	++
Lawton (2012) [42]	Y	Y	Y	Y	Y	Y	Y	Y	Y	U	Y	Y	Y	Y	++
Rankin (2012) [43]	Y	Y	Y	Y	Y	Y	Y	Y	Y	U	Y	Y	Y	Y	++
Rankin (2012) [44]	Y	Y	Y	Y	Y	Y	Y	Y	Y	U	Y	Y	Y	Y	++
Lawton (2013) [45]	Y	Y	Y	Y	Y	Y	Y	Y	Y	U	Y	Y	Y	Y	++
Rankin (2014) [46]	Y	Y	Y	Y	Y	Y	Y	Y	Y	U	Y	Y	Y	Y	++
Heller (2014) [47]	Y	Y	Y	Y	Y	Y	Y	Y	Y	U	Y	Y	Y	Y	++
REPOSE															
Lawton et al. (2014) [48]	Y	Y	Y	Y	Y	Y	Y	Y	Y	U	Y	Y	Y	Y	++
Heller et al. (2017) [49]	Y	Y	Y	Y	Y	Y	Y	Y	Y	U	Y	Y	Y	Y	++
DAFNE															
Snow et al. (2013) [50]	Y	Y	Y	Y	Y	Y	Y	Y	Y	Y	Y	Y	Y	Y	++
Snow et al. (2014) [51]	Y	Y	Y	Y	Y	Y	Y	Y	Y	Y	Y	Y	Y	Y	++
DAFNE-HART															
Shuttlewood et al. (2015) [52]	Y	Y	Y	Y	Y	Y	U	Y	Y	U	Y	Y	Y	Y	++
RAPID-CALC															
Knight et al. (2016) [53]	Y	Y	Y	Y	Y	U	Y	Y	Y	Y	Y	Y	Y	Y	++

++All or most of the checklist criteria have been fulfilled; where they have not been fulfilled the conclusions are very unlikely to alter. + some of the checklist criteria have been fulfilled, where they have not been fulfilled, or not adequately described, the conclusions are unlikely to alter. – Few or no checklist criteria have been fulfilled and the conclusions are likely or very likely to alter

*Y*: yes, *N*: no, *U*: unable to determine.

### The line of argument: Follow-up support for effective type 1 diabetes self-management (the FUSED model)

The line of argument synthesis resulted in the model presented below. The **F**ollow-**U**p **S**upport for **E**ffective type 1 **D**iabetes self-management (FUSED) model synthesises – or ‘fuses’ together – the learning from the included qualitative studies to outline the barriers to maintaining diabetes self-management practices after attending a SEP and presents the key features of follow-up support needed to facilitate effective, sustained disease self-management. The FUSED model describes how participants finish SEPs feeling enthusiastic about self-management practices, but subsequently, encounter various challenges to realising this in everyday life after course completion. The model outlines how participants respond to these challenges by adapting, simplifying, or personalising the self-management practices they were taught on SEPs. To help future participants sustain their learning and skills, the FUSED model proposes a combination of structured and responsive follow-up support guided by ten key recommendations. The model is presented diagrammatically in Fig. 2 and each of the concepts is described and explained below. For ease of reading, the diagram and narrative description have been organised into three sections: (A) Challenges encountered after course attendance; (B) Participant response to post-course challenges; (C) Recommendations for effective follow-up support. Table 6 also summarises how each included paper has contributed to the concepts within the line of argument.

**Table 6:** The parts making the whole - contribution of each study and paper to each concept in the line of argument

	STUDY:	Irish DAFNE			UK DAFNE									REPOSE		DAFNE		DAFNE-HART	RAPID CALC
	**PAPER:**	[36]	[37]	[38]	[39]	[40]	[41]	[42]	[43]	[44]	[45]	[46]	[47]	[48]	[49]	[50]	[51]	[52]	[53]
A. Challenges	Knowledgeable Empowered, Motivated	**x**	**X**	**x**	**x**	**x**	**x**	**x**	**x**	**x**	**x**	**x**	**x**		**x**	**x**	**x**	**x**	
	Complexity of Life	**x**		**x**	**x**		**x**						**x**			**x**			
	Disconnect between effort and reward	**x**		**x**		**x**			**x**							**x**	**x**		
	Lack of confidence in their own judgement		**x**					**x**		**x**			**x**	**x**					**x**
	Insufficient Support	**x**	**x**	**x**				**x**	**x**	**x**		**x**	**x**		**x**	**x**			
B. Participant response	Shift blood glucose targets			**x**					**x**	**x**	**x**		**x**						
	Stop or relax self-monitoring practices			**x**				**x**					**x**	**x**	**x**				**x**
	Over-rely on corrective doses							**x**					**x**						
	Over-treat hypoglycaemia										**x**		**x**					**x**	
	Simplify Life					**x**	**x**	**x**					**x**						
C. Follow-up	A combination of structured and responsive individual support	**x**						**x**		**x**		**x**	**x**			**x**		**x**	
	Modelling collaboration and empowerment		**x**		**x**			**x**										**x**	
	Preparing for and addressing motivational issues	**x**		**x**							**x**		**x**				**x**	**x**	
	Exploring and facilitating social support					**x**					**x**	**x**	**x**						
	Supporting the use of technology							**x**						**x**					**x**
	Educating mainstream health professionals									**x**			**x**			**x**			
	Building knowledge over time	**x**						**x**					**x**						
	Considering and revising routines and life circumstances						**x**												
	Reviewing and revising blood glucose monitoring and treatment practices			**x**				**x**	**x**										
	Reviewing and advising on hypoglycaemia management								**x**		**x**								**x**
	Providing dietary advice					**x**													

### A. Challenges encountered after course attendance

Armed with new knowledge and skills, participants leave SEPs feeling empowered and better equipped to undertake effective disease self-management [36, 47, 49–51]. They have a better understanding of their condition [36–38, 45, 50, 51] and having ‘rules’ to follow gives them greater confidence in undertaking self-management [39, 47]. Participants finish the course feeling motivated to put their new self-management skills into practice, driven by the desire to have less frequent hypoglycaemic episodes [36, 45], achieve tighter blood glucose control [39, 42, 43], prevent complications [36, 37], lower their HbA1c levels [37], and free themselves from previously restrictive treatment regimens [40, 41, 44]. Participants with impaired hypoglycaemia awareness similarly feel confident and committed to restoring awareness and implementing effective monitoring and treatment practices [52]. However, as we report below, after transitioning from the course into everyday life, all participants encounter various barriers to implementing their new knowledge and skills [47].

#### The complexity of life

Outside the cocooned classroom environment of SEPs, participants can feel trepidation about applying self-management practices in the complex contexts of their everyday lives [38, 39, 41, 50]. Participants feel most anxious in ‘untested’ or non-routine situations, such as when eating out [40, 47], at the weekend or on holiday [41]. Those who do not lead routinized lives with predictable working patterns and regular mealtimes can struggle to integrate self-management practices into everyday life and achieve stable blood glucose [39, 41, 47]. At critical junctures, when they face illness or bereavement for example, participants can intentionally or unintentionally prioritise other areas of their lives, resulting in less rigid application of self-management practices [36, 38, 47].

#### Disconnect between effort and reward

Participants want tangible rewards for their self-management efforts; for example, as already noted, a reduction in hypoglycaemic episodes or reduced HbA1c levels. However, when participants experience disconnect between effort and reward, they can become demotivated and frustrated. Repeatedly failing to achieve blood glucose levels within target ranges is demoralising [43, 51] and can result in feelings of failure [47], which counteracts participants’ initial feelings of motivation and empowerment [36]. Frustrations are even greater for participants whose glucose profiles fail to improve despite following self-management principles [36, 38], such as accurate carbohydrate counting which many find tedious [47]. Unanticipated consequences, such as gaining weight from greater dietary freedom, can also diminish the benefits of the new self-management approach [40].

#### Lack of confidence in their own judgement

Post-SEP many participants question their ability to review blood glucose readings, interpret patterns and make adjustments to background insulin doses and mealtime ratios [37, 42, 44, 47]. Participants may lack the confidence to calculate mealtime insulin doses [48] and be reluctant to change 1:1 mealtime insulin ratios as these keep their calculations simple [42, 47]. Although using an automated bolus advisor can help overcome these difficulties, over-reliance on this technology can prevent participants from developing their mathematical skills and taking greater control over self-management [48]. Some participants do have the confidence to over-ride the bolus advisor – for example, when undertaking planned physical activity or when there are technical problems. Some describe double-checking the bolus advisor’s recommendations by doing their own manual calculations [48, 53]. Most participants, however, simply trust the advisor without question [53] and many do not have the confidence or skills needed to change the settings independently in the event that their mealtime insulin requirements change [48]. Moreover, even with the assistance of a bolus advisor, many participants still prefer to defer self-management decisions to health professionals [42].

#### Insufficient support

While participants often struggle to adjust background insulin doses and mealtime ratios post-course [42, 43], many are reluctant to initiate contact with SEP educators for fear of over-burdening them [36, 44, 47]. Even when participants do initiate contact, educators may not be easily accessible and many wait until their next diabetes review appointment to raise questions [38, 44, 47]. Although participants may have other sources of support, these are not necessarily helpful. Many participants seek support from significant others [49], but friends and family often lack knowledge about diabetes management, leaving participants feeling confused as they attempt to implement new practices [48]. Following structured education, many participants find that they have greater condition-specific expertise than their primary care teams [44, 50]. Participants find it confusing and disheartening when health professionals ‘over-rule’ their newly acquired expertise, resulting in some avoiding contact with mainstream services altogether [37, 50].

### B. Participant response to challenges

As participants attempt to cope with the challenges they face post-SEP they:

#### Shift blood glucose targets

Many participants shift their blood glucose targets upwards over time, whether consciously or inadvertently [38, 43, 47]. This may be to make their targets more achievable or because they struggle to remember them, resulting in their re-instating those used pre-course [9]. Fear of hypoglycaemia, especially nocturnal episodes, can lead participants to use elevated targets as a safety net [38, 43, 45, 47]. As professional consultations typically focus on HbA1c rather than day-to-day blood glucose readings, participants’ misuse of targets may not always be identified and addressed in routine clinical care [47].

#### Stop or relax self-monitoring practices

Participants find recording and reflecting on their blood glucose readings burdensome, leading them to stop or relax these monitoring practices over time [38, 42, 47]. Even if participants continue to undertake regular self-monitoring of blood glucose, they may not make time for detailed data recording, reflection and looking for long term trends [42, 47]. Using an automated bolus advisor can reduce the burden of data recording [48, 49] and apps that link the bolus advisor to participants’ smartphones may be an even more convenient means of record keeping [53]. However, even when record keeping is easier, participants who use advisors may be less inclined to review blood glucose data than when they record it manually [48, 53].

#### Over-rely on corrective doses

Most participants find corrective doses easy to calculate and simple to use in addressing high glucose levels [42]. As a result, many come to rely predominantly on corrective doses to achieve target glucose levels rather than reviewing glucose profiles and using these to alter their background insulin doses or mealtime ratios [42, 47].

#### Over-treat hypoglycaemia

Some participants purposefully over-treat hypoglycaemia because they do not trust the treatment amounts specified on the course [45]. Participants who have had particularly traumatic hypoglycaemia experiences may over-treat as soon as they experience symptoms [47]. The panic, disorientation, lack of concentration and increased hunger during an episode can also lead to over-treatment [45, 47]. A few participants report using hypoglycaemia as an excuse to over-indulge in foods that they enjoy [45]. Moreover, over-treatment can arise when significant others (e.g. family members) feel panicked or lack knowledge of the correct treatment amounts [45, 47]. Although they are more likely to report better monitoring and treatment practices post-course, many of those with impaired hypoglycaemia awareness still find it difficult to set aside time to focus upon hypoglycaemia cues to improve their awareness [52].

#### Simplify life

Rather than being liberated from previous restrictive regimens, some participants instigate new, but similarly restrictive, practices and routines to help reduce the demands of a FIIT regimen, or increase chances of success [40–42, 47]. These include: regular mealtimes [41, 47]; choosing foods and portion sizes that make carbohydrate counting and mealtime insulin calculations easier and more accurate [41, 47]; avoiding snacks and, hence, the requirement to administer an insulin injection [40]; eating similar meals every day or ordering the same meal at a restaurant [40, 47]; and avoiding eating out [47]. In addition, participants may also rely on labelled, processed foods to make the determination of carbohydrate content easier [5, 12]. They may focus on carbohydrate rather than calorific content of foods resulting in inadvertent unhealthy food choices [47].

### C. Recommendations for effective follow-up support

To sustain effective self-management, participants require a combination of structured and responsive individualised follow-up support [36, 42, 44]. This is recommended for all participants, including those with optimal glycaemic control [36], who have access to good social support [46] and who report confidence in their knowledge and skills [50]. To address participants’ reluctance to over-burden health professionals, follow-up support should be fully integrated into the structured education package [44]. In addition to structured follow-up sessions, participants should be provided with opportunities for ad hoc contact with appropriately trained health professionals to troubleshoot problems as they arise and when life circumstances change [42, 44]. Although participants express a preference for receiving support from their own course educators [44, 52], they would also value input from other health professionals, providing they have specialised knowledge of FIIT [36, 42], either in person or via a dedicated phoneline [44, 47]. Although SEPs are effective in a group setting, following the SEP, participants indicate a clear preference for one-to-one, individually tailored follow up support [47]. The FUSED model recommends the following elements as the basis for effective follow-up support:

#### Modelling collaboration and empowerment

Follow-up sessions should take a collaborative approach to teaching, learning and problem solving that extends the rationale underpinning programmes by progressively inviting participants to take control, overcome deference to health professionals and bolster confidence in their own capability [37, 42, 52] to fit diabetes self-management into the complexity and stresses of modern life. For example, reviewing blood glucose data in a follow-up session should not focus solely on improving outcomes, but rather should seek to model the collaborative problem-solving process and use this as an opportunity to develop self-confidence and transform deferential attitudes [39]. The way information is presented and the interpersonal style of the healthcare professional delivering the support is key to promoting personal agency [52].

#### Preparing for and addressing motivational issues

Sustaining motivation is crucial if participants are to persist in using recommended self-management practices, particularly the more burdensome tasks such as record keeping [38]. Follow-up support can address motivational issues by pre-warning participants about and acknowledging feelings of disconnect between effort and reward [51] and ensuring ad-hoc support is readily available when needed [38]. Cognitive behavioural theory, motivational interviewing [52] and behaviour change theories [47] could also be used to inform the design of follow-up support to help promote sustained use of self-management practices. Course educators may benefit from additional psychological skills training [52] and could consider referring participants to specialist mental health input where appropriate [36, 45].

#### Exploring and facilitating social support

Asking participants about the level of support they receive from significant others (family, friends and work colleagues), how this helps or hinders self-management, and how they might draw upon social support in the future, can inform discussions and advice about how to effectively utilise social support [45, 46]. It may be appropriate to invite family members to attend specific follow-up sessions, for example on carbohydrate counting [47] or treating hypoglycaemia [46], or provide information and training geared specifically towards significant others [40, 45]. Any exploration of social support should be sensitive to participants’ preferences and the involvement of significant others should only be pursued if they think this would be helpful [46].

#### Supporting the use of technology

For participants who struggle to record, review and interpret their blood glucose readings, the development and use of technology, such as online diaries, apps or downloadable glucose meters, could make some self-management practices easier [42, 53]. Use of automated bolus advisors should be reviewed regularly at follow-up to ensure: the device is programmed with the correct parameters; participants understand how and when to change settings; and they continue to access and review their blood glucose data [48, 53]. If participants do not do this, follow up sessions could explore why and work with them to make more effective use of the technology [48]. Moreover, future technological advancements such as developing a bolus advisor or app that shows how the dose was calculated on screen might provide an important learning opportunity [53].

#### Educating mainstream health professionals

Dedicated follow-up support can prevent and counteract difficult interactions with non-specialist health professionals in mainstream services by being the first port of call for FIIT specific advice [44], providing training for participants in assertiveness skills, and writing letters to participants’ mainstream health professionals explaining the FIIT approach [50]. It is also crucial that all general health professionals are educated about self-management principles and how to develop collaborative working practices [47, 50].

#### Building knowledge and skills over time

Follow-up support provides an opportunity to review knowledge and skills post-course, highlight individual difficulties, revise knowledge and address skills gaps [36, 42, 47]. As well as helping to sustain knowledge and skills, follow-up support can build on and update participants’ knowledge in the months and years after course completion, for example by keeping them informed about new advances in knowledge [47]. This could involve providing information directly as part of follow-up sessions, or signposting to useful resources.

#### Considering and revising routines and life circumstances

Follow-up support should explore whether, and how, participants’ life circumstances, eating patterns and routines have changed post-course [41]. Participants could be advised to implement structured routines in the first instance, and then be supported to become progressively more flexible as they develop confidence and consolidate their self-management skills [41]. Highly routinized lifestyles are potentially detrimental to quality of life and could compromise participants’ motivation to sustain self-management principles; hence, participants should be encouraged to reflect on their satisfaction with routines [41].

#### Reviewing and revising blood glucose monitoring and treatment practices

Follow-up sessions should incorporate a review of blood glucose readings and support participants to consolidate their skills in identifying and interpreting patterns and responding with appropriate regimen changes [42]. It is important to reinforce the importance of record keeping in addition to regular blood glucose checking [38, 42], encourage participants to use clinically-recommended blood glucose target ranges, and help identify and address barriers to their use [42, 43].

#### Reviewing and advising on hypoglycaemia management

Follow-up support should review how participants are treating hypoglycaemia, identify whether they are over-treating and provide further education and advice where required [43, 45, 52]. Health professionals should review treatment of hypoglycaemia for all participants and not only those with identified impaired awareness of hypoglycaemia [43]. Follow-up support should explore with participants: their frequency of severe hypoglycaemic episodes; the blood glucose level they identify as ‘low’; and how often they have been prompted by significant others to treat hypoglycaemia [45]. Given that night-time targets are most likely to be adjusted upwards, follow-up support should include a conversation about attitudes towards nocturnal hypoglycaemia [43].

#### Providing dietary advice

During follow-up, facilitators need to establish if and what changes in food and eating practices have occurred as a result of attending the SEP to ensure that advice and support is correctly tailored. Follow-up sessions should: explore whether participants are restricting their food choices; identify those struggling with carbohydrate counting; explore if participants are relying more heavily on processed foods to aid carbohydrate counting and monitor their weight accordingly. Involving family or friends who influence food choices and preparation in the follow-up session, providing information to assist with knowing the carbohydrate and calorie content of foods, and appropriate referral to a dietician may be useful elements of a tailored approach for follow-up support [40].

## Discussion

Participation in a SEP, based on a FIIT approach, empowers individuals by providing them with the skills and competencies needed to undertake effective diabetes self-management. However, while participants who have attended a SEP often gain initial clinical benefit, typically evidenced by an improved HbA1c or a reduction in severe hypoglycaemic episodes, many encounter difficulties maintaining the skills and competencies taught on their courses over time. This is a pattern frequently shown in evaluations of self-management programmes, where benefits can be short-lived, and may attenuate substantially six months to a year after a programme ends [54, 55]. In this paper, which synthesises the available qualitative evidence about participants’ experiences of undertaking diabetes self-management after attending a SEP for people with T1DM, we add strength to the argument that skills training alone does not necessarily lead to individuals sustaining desired changes in behaviour. The FUSED model provides a framework for understanding why participants ***can*** struggle to maintain their learning and skills post-SEP attendance and, crucially, explicates how these barriers can be addressed through the provision of timely, tailored follow-up support.

The need for ongoing support for effective self-management following SEP is recognised not only for people with T1DM but also those with T2DM and other chronic health conditions [56–58]. While there is agreement that support needs to be ongoing and individualised, the actual features and components of such support and how it should be delivered are less well described. The FUSED model addresses this gap by outlining ten tangible recommendations for service delivery and acting as a logic model which can be used to underpin the design of follow-up support. Although developed in the context of T1DM, the model may also offer insights applicable to supporting self-management of other long-term chronic such as T2DM, asthma, cystic fibrosis.

The FUSED model calls for a more resource intensive and responsive approach which clearly may be more costly in the short-term than current approaches delivered as part of routine clinical care (e.g. annual/bi-annual diabetes review appointments). However, it may ultimately enable cost savings if it proves more clinically effective; for example, it may result in less patients progressing to requiring insulin pump therapy, and/or experiencing less diabetes-related microvascular complications. Before implementing such change in approach, it will be necessary to convince diabetes professionals of the potential benefits. Since many diabetes nurse specialists spend much of their time in repeated 1:1 consultations with individuals who are struggling to manage their diabetes successfully, which rarely leads to improved outcomes, they may be willing to explore different tactics. The multi-disciplinary team will also require additional training to deliver this new approach and finding trainers with the required skills and attitudes may be challenging. Hence, future work will be needed to establish the feasibility, acceptability and cost-effectiveness of a FUSED approach.

In addition, the model highlights the potential for technological support to sustain skills via online learning and enhanced monitoring. Existing research shows that technological support is likely to be most effective when it connects people with T1DM to their health care team using 2-way communication, where it analyses patient-generated health data, and provides tailored education and individualized feedback between a person with diabetes and their health care team [59]. As technology advances, it is crucial that future research explores how it can be successfully integrated within follow up support after SEP attendance to most effectively and efficiently provide the right support at the right time.

### What next?

We are using the FUSED model to inform the design of the follow-up support which will be delivered as part of a complex intervention called ‘DAFNE*plus*’. DAFNE*plus* is a structured education programme for people with T1DM, which, in addition to using a FUSED approach to follow-up support, comprises an adapted DAFNE course, embedding behaviour change/maintenance principles, and technological support. (https://dafneplusresearch.wordpress.com/).

DAFNE*plus* is currently being piloted in three sites prior to a definitive randomised controlled trial. The trial will include an economic and mixed-methods process evaluation which will allow us to explore how the FUSED model works in practice and evaluate this new approach to follow-up support. As well as looking at clinical and cost effectiveness, a key part of the research process will be to explore the acceptability of the FUSED follow-up model from both health professionals’ and participants’ perspectives. We will also determine how readily this new approach to follow-up support may be adopted into mainstream diabetes care.

### Strengths and weaknesses

Most of the papers included in the synthesis were of high quality and most reported longitudinal data collected at several time points up to a year following SEP attendance [36–47] with other papers reporting data from participants who had attended up to 11 years previously [50, 51]. The gaps and support requirements highlighted in the FUSED model are therefore supported by rich, long-term insights. When considering the potential limitations of this review, it should be borne in mind that the included papers explored the experiences of individuals who had attended the DAFNE programme in the UK and Southern Ireland. Hence, some recommendations may be less applicable in other countries where health care is funded and delivered differently and where other approaches to structured education are used. A further limitation is that, although the review draws on data from eighteen publications, these report from only six separate studies. There were also some limitations in the review process, with only one reviewer screening search results, and limited checking of data extraction raising the risk of error in identification of relevant studies and data. However, the involvement of four authors, who also were authors in 13 of the primary studies allowed interpretations of the data to be verified. To maintain rigor, these authors did not participate in quality appraisal of the included studies.

## Conclusion

In summary, our recommendation is that structured education should no longer be a discrete intervention, but, rather, an on-going programme involving a potentially life-long package of tailored and individualised follow-up support, which also uses technology to facilitate and support those close communications with specialist health professionals and builds self-efficacy to confidently maintain self-management. Using a meta-ethnographic approach to a systematic review of the qualitative literature of people’s long-term experiences of self-management in T1DM, we have developed the FUSED model to guide the development of follow-up support for adults with T1DM. The model can contribute to the redesign of patient-centred health services, HCP training, and availability of technology. It may also provide insights for other conditions, which also rely on long-term self-management.

## Additional files

Additional file 1: Sample search strategy. (PDF 95 kb)

Additional file 2: List of excluded studies. (PDF 42 kb)

## Abbreviations

BME: Black and minority ethnic
DAFNE: Dose Adjustment for Normal Eating
DAFNE-HART: DAFNE + Hypoglycaemia Awareness Restoration Therapy
DTTP: Düsseldorf, the Diabetes Treatment and Teaching Programme
FIIT: Flexible intensive insulin therapy
FUSED: Follow-Up Support for Effective type 1 Diabetes self-management
HbA1c: Glycated haemoglobin
REPOSE: Relative Effectiveness of Pumps Over MDI and Structured Education
SEPs: Structured education programmes
T1DM: Type 1 diabetes mellitus
T2DM: Type 2 diabetes mellitus
