# Supplementary material for: Follow-Up Support for Effective type 1 Diabetes self-management (The FUSED Model): A systematic review and meta-ethnography of the barriers, facilitators and recommendations for sustaining self-management skills after attending a structured education programme
Source: BMC Health Serv Res. 2018 Nov 27;18:898. doi: 10.1186/s12913-018-3655-z (PMC6258400; doi:10.1186/s12913-018-3655-z)
Supplement: Supplementary file 1 — Sample search strategy. (PDF 95 kb) [file 12913_2018_3655_MOESM1_ESM.pdf]

Additional file 1

EXAMPLE SEARCH STRATEGY

Database: Ovid MEDLINE(R) Epub Ahead of Print, In-Process & Other Non-Indexed Citations, Ovid MEDLINE(R) Daily and Ovid MEDLINE(R) <1946 to Present>

Search Strategy:

----

1 Diabetes Mellitus, Type 1/
2 Diabetic Ketoacidosis/
3 (IDDM or T1DM or T1D).tw.
4 (("insulin* depend*" or "insulin?depend*") not ("non-insulin* depend*" or "noninsulindepend*")).tw.
5 (("typ? 1" or "typ? I" or "typ?1" or "typ?I") adj2 diabet*).tw.
6 ((Sudden-onset or sudden onset or ketosis-prone or ketosis prone) adj3 diabet*).tw.
7 ((acidos* or keto* or labil* or britt*) adj3 diabet*).tw.
8 ((Auto-immun* or autoimmun*) adj3 diabet*).tw.
9 (insulin* defic* adj2 absolut*).tw.
10 Hyperglycemia/ or Hypoglycemia/
11 (Hyperglyc?emia or hypoglyc?emia).tw.
12 Blood Glucose/
13 Glycemic Index/
14 (Glyc?emic adj (index or load)).tw.
15 1 or 2 or 3 or 4 or 5 or 6 or 7 or 8 or 9 or 10 or 11 or 12 or 13 or 14
16 (DAFNE or BERTIE).tw.
17 exp Self Care/
18 *Self Efficacy/
19 (Self adj (manage* or monitor* or care or efficacy or regulat* or empower* or control*)).tw.
20 ((Behavio?r or lifestyle) adj3 (change or modif* or alter* or intervention* or technique*)).tw.
21 *Adaptation, Psychological/
22 (Adapting or adaptation or coping).tw.
23 *Goals/
24 Goal setting.tw.
25 *Motivation/
26 Goal setting.tw.
27 *Motivational Interviewing/
28 (Motivational adj (counselling or interview*)).tw.
29 *Health Education/
30 *Patient Education as Topic/
31 ((Health or patient) adj2 (educat* or learning)).tw.

32 (Shared adj3 (learn* or support* or program* or tutor* or advice or monitor* or train* or instruct* or consult* or educat*)).tw.
33 (Face-to-face adj3 (learn* or support* or program* or tutor* or advice or monitor* or train* or instruct* or consult* or educat*)).tw.
34 (Community adj3 (learn* or support* or program* or tutor* or advice or monitor* or train* or instruct* or consult* or educat*)).tw.
35 (Individual* adj3 (learn* or support* or program* or tutor* or advice or monitor* or train* or instruct* or consult* or educat*)).tw.
36 *Peer group/
37 (Peer* adj3 (learn* or support* or program* or tutor* or advice or monitor* or train* or instruct* or consult* or educat*)).tw.
38 (Group adj3 (learn* or support* or program* or tutor* or advice or monitor* or train* or instruct* or consult* or educat*)).tw.
39 *Social Support/
40 (Social adj3 (learn* or support* or program* or tutor* or advice or monitor* or train* or instruct* or consult* or educat*)).tw.
41 ((Booklet* or leaflet* or pamphlet* or information) adj3 (patient? or care or health* or behavio?r or lifestyle* or diet*)).tw.
42 *Health Promotion/
43 ((Health* adj promot*) or adviser* or advisor*).tw.
44 Patient Compliance/
45 intensive insulin therapy.mp.
46 17 or 18 or 19 or 20 or 21 or 22 or 23 or 25 or 26 or 27 or 28 or 29 or 30 or 31 or 32 or 33 or 34 or 35 or 36 or 37 or 38 or 39 or 40 or 41 or 42
47 Reminder Systems/
48 (Reminder* or prompt*).tw.
49 Telemedicine/
50 (Telemedicine or telecommunication or telecare or telemonitor*).tw.
51 (Tele-medicine or tele-communication or tele-care or tele-monitor*).tw.
52 exp computer communication networks/
53 Hypermedia/
54 exp Cell Phones/
55 Internet/
56 Telephone/
57 (Internet or online or blog* or wiki or "social media" or "social network*" or Facebook or Twitter or automat* or "virtual communit*").tw.
58 (Computer* or hypermedia* or "web*based").tw.
59 (Telephone* or cellphone* or cell-phone* or smartphone* or smart-phone* or "mobile phone*").tw.
60 Computers, Handheld/

61 Multimedia/
62 Video Recording/
63 (Video* adj3 (record* or conference* or instruct* or teach* or learn* or support*)).tw.
64 (SKYPE or "audiovisual dialog*" or "audio-visual dialog*").tw.
65 (E-tool$1 or e-health$ or e-medicine or e-practice or e-instruct*).tw.
66 or/47-65
67 16 or (46 and 66)
68 grounded theory/
69 grounded theory.mp.
70 phenomenolog*.mp. [mp=title, abstract, original title, name of substance word, subject heading word, keyword heading word, protocol supplementary concept word, rare disease supplementary concept word, unique identifier, synonyms]
71 ethnograph*.mp. [mp=title, abstract, original title, name of substance word, subject heading word, keyword heading word, protocol supplementary concept word, rare disease supplementary concept word, unique identifier, synonyms]
72 Interview/
73 interview.tw.
74 focus groups/ or interviews as topic/
75 focus group.tw.
76 observation.tw.
77 Observation/
78 questionnaire*.tw.
79 thematic analysis.mp.
80 constant comparison.mp.
81 content analysis.mp.
82 themes.tw.
83 category.tw.
84 (experience or views or experiences or perspective*).mp. [mp=title, abstract, original title, name of substance word, subject heading word, keyword heading word, protocol supplementary concept word, rare disease supplementary concept word, unique identifier, synonyms]
85 68 or 69 or 70 or 71 or 72 or 73 or 74 or 75 or 76 or 77 or 78 or 79 or 80 or 81 or 82 or 83 or 84
86 15 and 67
87 interview:.tw. or px.fs. or exp health services administration/
88 (qualitative or themes).tw.
89 (interview: or experience:).mp. or qualitative.tw.
90 87 or 88 or 89
91 85 or 90
92 86 and 91
93 limit 92 to yr="1978 -Current"

94 86 and 90
