# Supplementary material for: Follow-Up Support for Effective type 1 Diabetes self-management (The FUSED Model): A systematic review and meta-ethnography of the barriers, facilitators and recommendations for sustaining self-management skills after attending a structured education programme
Source: BMC Health Serv Res. 2018 Nov 27;18:898. doi: 10.1186/s12913-018-3655-z (PMC6258400; doi:10.1186/s12913-018-3655-z)
Supplement: Supplementary file 2 — List of excluded studies. (PDF 42 kb) [file 12913_2018_3655_MOESM2_ESM.pdf]

Additional file 2
Table of Excluded Citations

**Author**	***Date***	***Title***	**Include/exclude**	***Reason for exclusion***
Beverly	*2013*	*Explaining Patients' Responses to Diabetes Education: A Qualitative Study.* Diabetes et al.	exclude	T1DM and T2DM
Bianchi	*2011*	*Motivating type 1 diabetic patients to learn carbohydrate counting through a cultural/aesthetic experience.* Diabetologia et al.	abstract	Insufficient data
Buysschaert	*1987*	*Effect of an inpatient education programme upon the knowledge behaviour and glycemic control of insulin-dependent diabetic patients.* . Diabetes & Metabolism et al.	exclude	Quantitative study
Cagle	*2002*	*Practitioners' actions inhibited patient participation in self care decision making.* Evidence Based Nursing	exclude	Not participated in a SEP
Cameron	*2016*	*Self-monitoring of blood glucose in insulintreated diabetes: A case study of patients* and F. Harris J.M.M. Evans	exclude	Not participated in a SEP
Cheyette	*2012*	*Early changes in glycated haemoglobin (HbA1c) can be used to predict glycaemic outcomes at 1 year following the Dose Adjustment for Normal Eating (DAFNE) course.* Diabetic Medicine	exclude	Quantitative study
Cooke	*2013*	*Linguistic and psychometric validation of the Diabetes-Specific Quality-of-Life Scale in U.K. English for adults with type 1 diabetes.* Diabetes care et al.	Exclude	Not a qualitative study
Davis	*2007*	*Social Identity Theory and Telehealth: A New Behavioral Approach to Frame Diabetes Self-Management.* Diabetes	abstract	Insufficient data
Davis	*2012*	*Identifying provider and patient viewpoints in developing a diabetes selfmanagement guide.* Journal of Investigative Medicine et al.	exclude	T2DM
Fairfield	*2014*	*Getting there': The impact of structured insulin management education in a high ethnic mix population with type 1 and type 2 diabetes.* Practical Diabetes and J. James S. Amin	exclude	Quantitative study
Fortin	*2017*	*Practices perceptions and expectations for carbohydrate counting in patients with type 1 diabetes - Results from an online survey.* Diabetes Research & Clinical Practice et al.	exclude	Web based questionnaire
Gilbert	*2012*	*Online communities are valued by people with type 1 diabetes for peer support: How well do health professionals understand this?* Diabetes Spectrum et al.	exclude	Not attended a SEP
Glindorf	*2012*	*Effect of a 5 days intervention programme for people with type 1 diabetes and severe hypoglycaemia.* Diabetologia	abstract	Insufficient data
Gundupalli	*2010*	*Effect of Family Support and Education on Diabetes Self Management in Cuban Americans.* Faseb Journal and F.G. Huffman J. Vaccaro	abstract	Insufficient data

Harris	*2017*	*Barriers to attendance at Dose Adjustment for Normal Eating (DAFNE) in South London: A qualitative study.* Diabetic Medicine and S.A. Amiel H. Mulnier	exclude	Postal/screening survey*
Harris	2016	Marketing Dose Adjustment For Normal Eating (DAFNE): Exploring perceived barriers to attendance in Healthcare professionals (HCP) and adults with Type 1 diabetes. Diabetic Medicine et al.	exclude	Not attended a SEP
Harry	*2013*	*[Time and use of discussion forums in type 1 diabetes: contribution to patient education].* Temporalite et usage des forums asynchrones dans le diabete de type 1. Contribution a l'Education therapeutique du patient.	exclude	Not participated in SEP
Helme	*2004*	*Patient accounts for noncompliance with diabetes self-care regimens and physician compliance-gaining response.* Patient education and counseling	exclude	surveys
Hinder	2012	"This does my head in". Ethnographic study of self-management by people with diabetes. BMC health services research	exclude	T1DM and T2DM
Kramer	*2013*	*Which principles patients with diabetes type 1 use for self-adjustment of insulin dose?* Diabetologia et al.	abstract	Insufficient data
Lehmann	*2000*	*User experience with the AIDA interactive educational virtual diabetes patient simulator.* Diabetes technology & therapeutics	absract	Not participated in a SEP
Lucherini	*2016*	*Performing Diabetes: Felt Surveillance and Discreet Self-Management.* Surveillance & Society	exclude	Not participated in SEP
McIntyre	*2014*	*Dose adjustment for normal eating: A role for the expert patient?* Diabetes and Metabolism Journal	exclude	Descriptive study
McIntyre	*2006*	*DAFNE (Dose Adjustment for Normal Eating): structured education in insulin replacement therapy for type 1 diabetes.* The Medical journal of Australia	exclude	editorial
Mullan	*2012*	*Exploring the experiences of adults living with type 1 diabetes accessing care.* Canadian Journal of Diabetes et al.	exclude	Not participated in a SEP
O'Hara	2013	A cross country qualitative investigation of structured education programmes for adults with type 1 diabetes. Diabetologia	exclude	abstract
O'Kane	*2016*	*Individual differences and contextual factors influence the experience and practice of self-management with type 1 diabetes technologies.* Diabetes Technology and Therapeutics	exclude	Not participated in a SEP
Oliver	*2009*	*The DAFNE Collaborative: Experiences of developing a nationally delivered evidence-based quality-assured programme for people with type 1 diabetes.* Practical Diabetes International	exclude	Not a qualitative study

Oliver	*2011*	*Carbohydrate counting in diabetes.* Nursing times	exclude	editorial
Ozcan	2012	Redesigning an intensive insulin service for patients with Type 1 diabetes: A patient engagement exercise. Diabetic Medicine et al.	exclude	Not attended a SEP
Rasmussen	*2007*	*Young women with diabetes: using Internet communication to create stability during life transitions.* Journal of P. Dunning	exclude	Not attended a SEP
Richard	*1989*	*ATTITUDES OF INSULIN-DEPENDENT DIABETICS TOWARDS THEIR DISEASE FOLLOWING A ONE-WEEK EDUCATION-PROGRAM.* Semaine Des Hopitaux	exclude	French language
Rosenbek Minet	2011	The experience of living with diabetes following a self-management program based on motivational interviewing. Qualitative health research et al.	exclude	Not all had attended a SEP
Sacco	*2012*	*Educational attainment moderates the effect of a brief diabetes self-care intervention.* Diabetes research and clinical practice et al.	exclude	Not part of a SEP
Savin	*2013*	*Empowering young women with type 1 diabetes: Evaluating a peer-led diabetes support group.* Diabetes	exclude	Not participated in a SEP
Schaefer	2014	Strategies for Improving Participation in Diabetes Education. A Qualitative Study. Plos One et al.	exclude	Not attended a SEP
Shepard	*2012*	*Patient perspectives on personalized glucose advisory systems for type 1 diabetes management.* Diabetes technology & therapeutics et al.	exclude	Not participated in a SEP
Simon	*2014*	*Diabetes patients' experiences with the implementation of insulin therapy and their perceptions of computer-assisted self-management systems for insulin therapy.* Journal of medical Internet research	exclude	T2DM
Stadler	2014	DAFNE-HART a psycho-educational programme to reverse hypoglycaemia unawareness in Type 1 diabetes: Report on sustained biomedical benefit at 1 year	exclude	Quantitative results
Storni	*2014*	*Diabetes self-care in-the-wild: Design challenges for personal health record systems and self-monitoring technologies.* Information Technology & People	exclude	No evidence of attending a SEP
Trief	*2003*	*Describing support: A qualitative study of couples living with diabetes.* Families e	exclude	No evidence of attending a SEP
Waite	*2013*	*Mobile phone applications and type 1 diabetes: An approach to explore usability issues and the potential for enhanced self-management.* Diabetes & Primary Care	exclude	No evidence of SEP
Willaing	*2013*	*Exploring diabetes education and information: perspectives of people with diabetes (DAWN2 study).* Diabetologia	exclude	Abstract, T1DM and T2DM
Wilson	2009	Behavioural change in type 1 diabetes self-management: Why and how? Health Education Journal	include	Not attended a SEP teaching FIIT
